# Generating a Preclinical Model for PITPNM3 and Evaluating Genotype–Phenotype Concordance: Insights from a Mouse Model

**DOI:** 10.3390/cells14201626

**Published:** 2025-10-18

**Authors:** Aykut Demirkol, Joanne Li, Stephen H. Tsang

**Affiliations:** 1 Jonas Children’s Vision Care and Bernard & Shirlee Brown Glaucoma Laboratory, Institute of Human Nutrition, Columbia Stem Cell Initiative, New York, NY 10032, USA; ad3871@cumc.columbia.edu (A.D.); sht2@cumc.columbia.edu (S.H.T.); 2 Edward S. Harkness Eye Institute, Columbia University Irving Medical Center, New York-Presbyterian Hospital, New York, NY 10032, USA; 3 Edward S. Harkness Clinical Coordinating Center, Columbia University, New York, NY 10032, USA; 4 Department of Clinical Sciences, Kaiser Permanente Bernard J. Tyson School of Medicine, Pasadena, CA 91101, USA; 5 Department of Biomedical Engineering, Columbia University, New York, NY 10027, USA; 6 Departments of Ophthalmology, Pathology & Cell Biology, Vagelos College of Physicians and Surgeons, Columbia University Irving Medical Center, New York, NY 10032, USA

**Keywords:** PITPNM3 preclinical model, genotype–phenotype concordance, retinal function, mouse model

## Abstract

PITPNM3 has been identified as a crucial gene associated with various phenotypes of retinal disease in humans; however, detailed mechanisms through which PITPNM3 mutations result in these conditions are not fully understood. In this study, we aimed to generate such a preclinical mouse model and evaluate its relevance to human PITPNM3-related conditions. Heterozygous mice were bred to obtain a homozygous genotype, aiming to mimic the human genetic condition. Subsequent phenotyping and genetic segregation analyses were conducted along with electrophysiological studies and histological examinations. Full-field electroretinogram analysis revealed a reduced cone response although the severity was not as pronounced as observed in humans with PITPNM3-related conditions. Histologically, the retinal structure appeared largely unchanged, indicating a discordance between functional impairment and morphological changes. In our preclinical mouse model, the observed phenotypic changes were not as severe as those found in humans with PITPNM3-related conditions and this discrepancy points to a potentially different disease progression trajectory in the mouse model. These findings highlight the importance of longer follow-up periods in such studies and the need for further research to elucidate the genotype–phenotype relationship in PITPNM3.

## 1. Introduction

The comprehension of genetic contributions to human diseases has experienced significant advancements in recent years. What was once purely theoretical, gene therapy has now materialized as a viable treatment for conditions like primary immunodeficiency and hemophilia, with numerous ongoing clinical trials [1,2]. Gene therapy has particularly focused on monogenic diseases with recessive inheritance, given the straightforward nature of the disease-causing genotype [3]. The retina stands out as a distinctive target for gene therapy due to its immunoprivileged environment, the convenience of subretinal injection, and direct visibility [4].

One gene that has gained significant attention in the field of genetics is PITPNM3, also known as phosphatidylinositol transfer protein membrane-associated 3. A 20-exon gene spanning 101 kb on human chromosome 17p13 [5], PITPNM3 codes for calcium-binding proteins with phosphatidylinositol transfer ability [6], and plays a crucial role in various cellular processes, including signal transduction, vesicle trafficking, and membrane lipid metabolism [7]. PITPNM3 homologues have long been tied to phototransduction in Drosophila and rat models [5,8]. More recently, PITPNM3 mutations have been implicated to several human disorders, particularly those affecting the retina, such as retinitis pigmentosa (RP) [9], and cone–rod dystrophy [10].

Previous reports have linked PITPNM3 to autosomal dominant cone-rod dystrophy (CORD5) [11]. The locus of CORD5 spans a region of 14.3cM and encompasses PITPNM3 as well as AIPL1 and GUCY2D [11]. Distinguishing itself from other autosomal dominant CORDs, CORD5 is restricted to loss of cone photoreceptor function and appears to spare rod photoreceptors [10]. Mutations in neighboring gene GUCY2D have been shown to be implicated in CORD5 as well as Leber congenital amaurosis [12]. Case studies show PITPNM3-associated CORD5 has a varied phenotypic presentation. In one family with identified PITPNM3 mutation, patients presented with reduced visual acuity and light sensitivity from childhood, progressing to legal blindness by early adulthood, while patients in another family showed more mild symptoms with later onset in adulthood [10].

Mutations in PITPNM3 have also been implicated as a cause of autosomal recessive retinitis pigmentosa (RP). RP is a group of inherited retinal dystrophies characterized by progressive degeneration of photoreceptor cells, leading to visual impairment and eventual blindness [13]. The mechanism by which PITPNM3 mutations contribute to RP pathogenesis is not fully understood, but PITPNM3′s involvement in lipid metabolism and intracellular signaling pathways may play a possible role. Notably, a missense mutation in PITPNM3 was found in a case report of a patient with autoimmune retinopathy presenting with rod-cone dystrophy and rapidly deteriorating vision [9]. The involvement of PITPNM3 in numerous retinopathies with high phenotype variation necessitates further investigation.

Preclinical models have been employed to better understand the functional consequences of PITPNM3 mutation and genotype–phenotype interactions. Previous preclinical models have shown that the drosophila homologue of PITPNM3, rdgB, is involved in photoreceptor response and prevention of retinal degeneration [8]. Expression of PITPNM3 homologues in the retina differs by species. In zebrafish, expression in the retina is limited to cone cell inner segments [14]. Expression of the rat homologue shows variation by stage of development, strongly expressed in the ganglion cells and outer retina early on and settling in the inner segment in adulthood [15]. Mouse models have shown that PITPNM3 is expressed in retinal outer nuclear segments [16].

Despite the progress made in understanding the role of PITPNM3 in human conditions through preclinical models, there are still significant gaps in knowledge that warrant further investigation. First, we have limited understanding of the exact molecular mechanisms by which PITPNM3 mutations lead to retinal degeneration. While studies have implicated lipid metabolism and intracellular signaling pathways [7], further research is needed to unravel the precise interactions and downstream effects of PITPNM3 dysregulation. Second, there is incomplete genotype–phenotype concordance observed between PITPNM3-related phenotypes in humans and existing preclinical models. Thus, there is a need to develop preclinical models that better recapitulate the human disease phenotype in severity and progression of retinal degeneration and allow for a comprehensive assessment of the genotype–phenotype relationships.

The current study aims to address these gaps by developing a more refined preclinical model in mice for studying PITPNM3-related phenotypes. By bridging the gap between genetic variants and clinical manifestations, the study seeks to generate a more accurate representation of the human condition and pave the way for development of targeted therapeutic interventions.

## 2. Materials and Methods

### 2.1. Generation and Genotyping of PITPNM3 Mouse Lines

To establish a mouse model that closely parallels the human genetic context of PITPNM3-related retinal disease, heterozygous mice carrying PITPNM3 mutations were bred together. This approach aimed to generate offspring that were homozygous for the PITPNM3 mutation, thereby recapitulating the biallelic loss-of-function scenario found in patients with severe forms of the disease. All mice were housed and cared for in a controlled environment designed to minimize extraneous variables, thus ensuring consistency and reliability in both animal health and experimental outcomes. Following breeding, genomic DNA was extracted from tail biopsies of each mouse.

The PITPNM3 mutant mouse line carries a targeted knock-in of the V367M variant, corresponding precisely to a missense mutation (c.1099G>A, p.Val367Met) previously described in human patients with autosomal dominant cone-rod dystrophy (CORD5) [REF]. This mutation site was confirmed by Sanger sequencing in all experimental animals. We chose this humanized mutation to maximize translational relevance between the model and patient phenotype. PITPNM3 expression, as assessed by in situ hybridization and immunostaining, is highest within rod and cone photoreceptors, as well as the retinal pigment epithelium. Thus, affected cell types in our model include both photoreceptors and RPE, as in human disease. The precise genotype was confirmed through polymerase chain reaction (PCR) amplification and Sanger sequencing, verifying the presence and zygosity of the intended PITPNM3 mutation. This rigorous screening process was crucial in attributing observed phenotypes solely to alterations in PITPNM3, effectively eliminating potential confounders arising from genetic drift or background mutations.

### 2.2. Electroretinography (ERG)

Functional assessment of retinal integrity was conducted using full-field electroretinography (Appendix A.1), a highly sensitive technique to measure the electrical responses of photoreceptors and downstream retinal cells to light stimulation. Mice were dark-adapted overnight to maximize retinal responsiveness and then anesthetized to ensure immobilization during data acquisition. Gold wire or contact lens electrodes were gently placed on the surface of each cornea, while reference and ground electrodes were positioned subcutaneously at predetermined sites. Mice were then exposed to a stepwise protocol of light flashes spanning a range of intensities. As previously described, pulses of 0.00130 cd/m^2^ and 3 cd/m^2^ (White-6500K) were employed [17,18,19]. This allowed the specific testing of rod and cone functionality so that the individual and combined function could be analyzed. Retinal responses were recorded as waveforms consisting of an initial negative deflection (A-wave, photoreceptor response) and subsequent positive deflection (B-wave, reflecting activity of bipolar and Müller cells). Both the amplitude and the latency of these signals were analyzed. It was thus possible to detect even subtle functional deficits in retinal processing, providing detailed functional genotype–phenotype correlation and facilitating comparisons to clinical findings in human PITPNM3-associated retinopathies.

### 2.3. Histological Analysis

**Figure A1:**
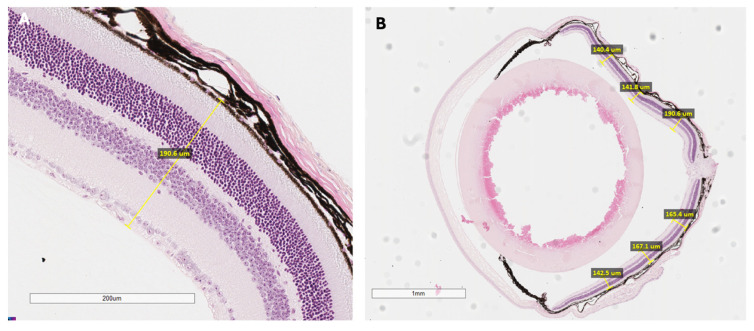
Example of retina thickness measurement from the PITPNM3 heterozygous, RD1 wild-type mouse line. (**A**) Measurement taken from inner border of the internal limiting membrane to the outer border of retinal pigmented epithelium, with (**B**) measurements taken at 1500, 1000, 500, −500, −1000, and −1500 from the optic nerve in each mouse.

Subsets of mice underwent histological analysis to directly examine microscopic retinal structure and cellular organization (Appendix A.2). After functional testing, mice were euthanized, and eyes were promptly enucleated. Each globe was fixed in paraformaldehyde to preserve tissue architecture, embedded in paraffin, and serially sectioned at defined thicknesses suitable for mouse ocular tissue. These sections were then stained, typically with hematoxylin and eosin, to allow for clear visualization of individual retinal layers under light microscopy. Particular attention was paid to the thickness and organization of the outer nuclear layer, the integrity of photoreceptor cells, and the structure of the retinal pigment epithelium (RPE). Quantitative measurements of retinal thickness were obtained at standard positions relative to the optic nerve head, which permitted precise spatial mapping of degenerative or dysplastic changes (Figure A1). This meticulous approach enabled a robust structural assessment, complementing the functional and imaging-based findings in the study.

### 2.4. Retinal Phenotyping in PITPNM3 Mouse Model and Human Disease

Comprehensive retinal imaging and functional analyses were performed to characterize the effects of PITPNM3 mutations in both the preclinical mouse model and human patients. This approach integrated color fundus photography, fundus autofluorescence (FAF), optical coherence tomography (OCT), and histological assessment in the animal model for robust genotype–phenotype correlation.

### 2.5. Integrated Rationale for Multimodal Phenotyping

Employing this multimodal, layered phenotyping strategy—combining quantitative imaging, electrophysiological measurement, and direct histological examination—ensured a comprehensive and nuanced characterization of PITPNM3-related retinal disease in the mouse model. This design enabled the detection not only of overt manifestations but also of subtle, incipient changes that precede clinically significant degeneration. Through detailed genotype–phenotype correlation, the approach facilitated benchmarking of the mouse model against clinical features observed in human patients, enhancing our understanding of disease mechanisms and progression. All animal procedures conformed to ethical guidelines promulgated by institutional review boards and adhered strictly to best practices for animal research.

## 3. Results

### 3.1. Patient Retinal Imaging

**Figure 1:**
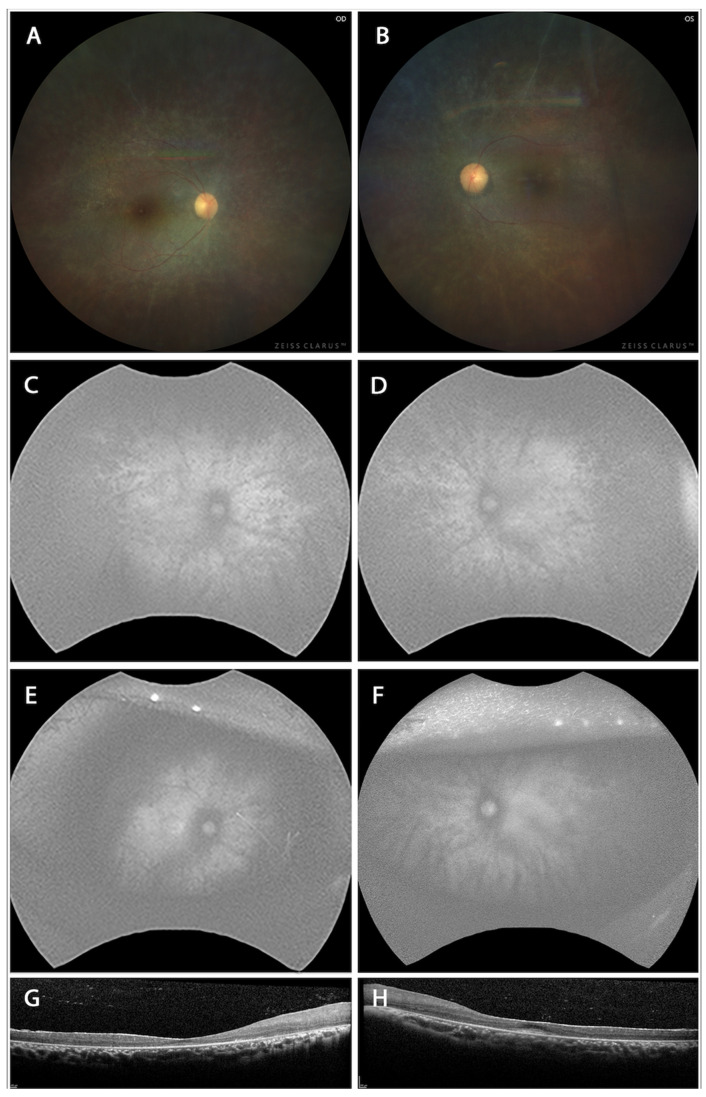
Color fundus photographs of the right (**A**) and left (**B**) eyes show optic disc pallor, generalized attenuation of retinal arterioles, and a granular, mottled appearance of the peripheral retina with subtle pigmentary changes. Fundus autofluorescence (FAF) images of the right (**C**,**E**) and left (**D**,**F**) eyes at two time points demonstrate a widespread, speckled pattern of hypoautofluorescence extending from the posterior pole to the mid-periphery suggest. Spectral-domain optical coherence tomography (SD-OCT) of the right (**G**) and left (**H**) eyes reveals thinning of the outer retina with attenuation or loss of the ellipsoid zone outside the foveal center.

Color fundus photographs in Figure 1 are derived from two unrelated patients known to harbor the PITPNM3 V367M mutation, confirmed by Sanger sequencing of genomic DNA. These images are intended to serve as clinical comparators for the phenotypes observed in our genetically engineered mouse model. Color fundus photographs of human patients (Figure 1A,B) revealed optic discs with mild pallor, generalized narrowing of retinal arterioles, and a peripheral granular, mottled appearance with subtle pigmentary changes. Notably, classic “bone spicule” pigmentation was absent, and the macula displayed relative sparing with loss of the normal foveal reflex. These features align with a rod-cone dystrophy such as retinitis pigmentosa sine pigmento, where the absence of bone spicules signals an early or atypical disease stage.

Fundus autofluorescence (FAF) images of the right (Figure 1C,E) and left (Figure 1D,F) in patients demonstrated a widespread, speckled hypoautofluorescence pattern radiating from the posterior pole to the periphery. Central macular autofluorescence was relatively preserved, encircled by an abnormality ring, and the pattern demonstrated symmetry between the eyes. These findings point to widespread RPE dysfunction or atrophy characteristic of panretinal dystrophy, but with spared central vision. In the PITPNM3 mouse model, both heterozygous and homozygous mutants maintained comparable quantitative autofluorescence (qAF) levels to wild-type controls early on, reinforcing the notion that RPE pathology in mice develops gradually and may require longer observation for overt changes to manifest.

Spectral-domain OCT of the right (Figure 1G) and left (Figure 1H) disclosed thinning of the retinal layers, especially the outer retina, and loss or attenuation of the ellipsoid zone beyond the foveal center. Importantly, the foveal contour was preserved, and there was no evidence of cystoid macular edema or subretinal fluid. Such findings are common in inherited retinal degenerations and reflect progressive photoreceptor loss with central preservation in early to moderate stages.

### 3.2. Electroretinography

**Figure 2:**
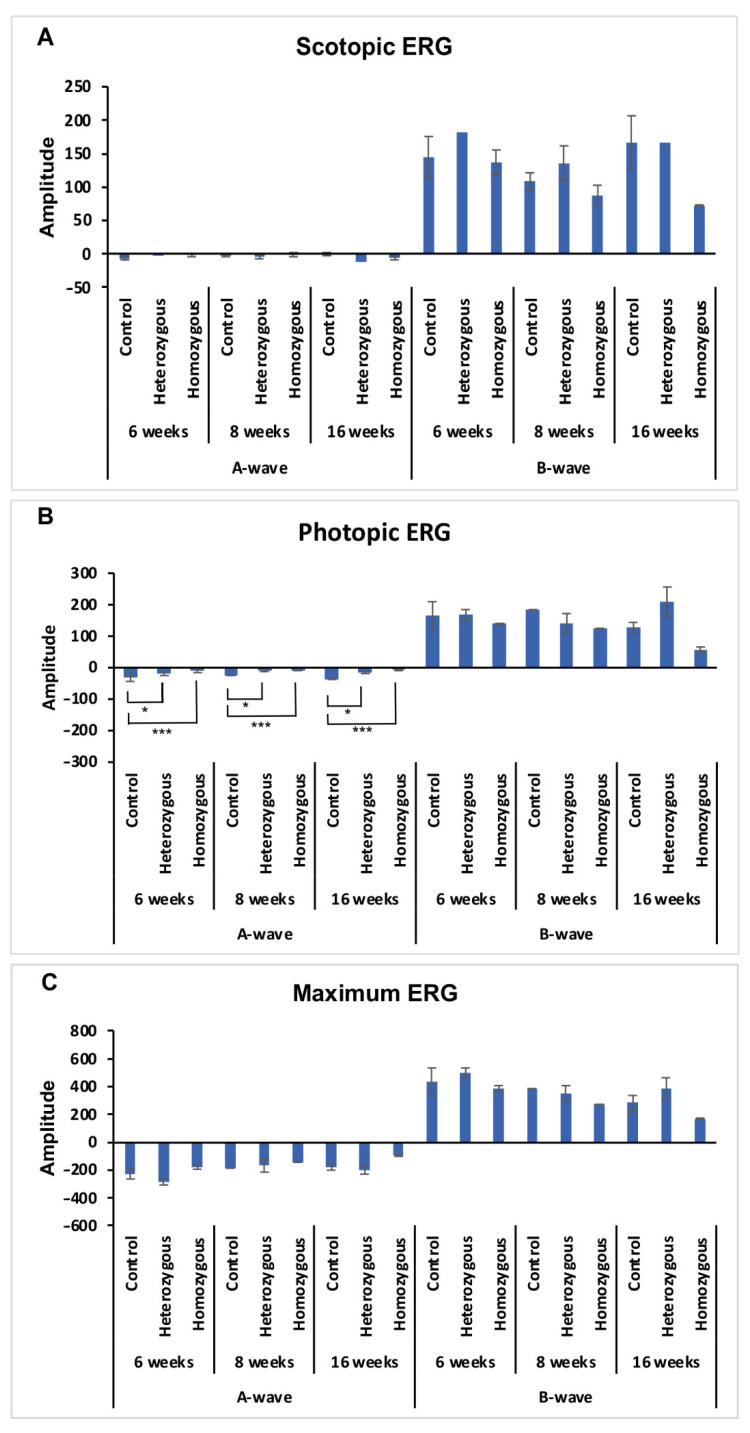
ERG amplitudes by PITPNM3 genotype under scotopic, photopic, and maximum stimulus conditions. Data are mean ± SEM for control, heterozygous, and homozygous mice at each week. Pairwise comparisons within each week were by Tukey adjusted ANOVA; * *p* < 0.05, *** *p* < 0.001 versus wt. (**A**) Left: Scotopic a-wave amplitudes did not differ by genotype (mutation *p* = 0.892). Right: Scotopic b wave amplitudes were also similar across genotypes (mutation *p* = 0.693). (**B**) Left: Photopic a-waves differed significantly by genotype (mutation *p* = 0.00024, Levene’s *p* = 0.0097); Heterozygous vs. Control, * *p* = 0.034; Homozygous vs. control, *** *p* = 0.0002. Right: Photopic b-waves were similar across genotypes (mutation *p* = 0.224). (**C**) Left: a-waves did not differ by mutation (*p* = 0.421). Right: b-waves were also non-significant (*p* = 0.851). Age was included as a covariate in all models but was never significant for these endpoints.

**Figure 3:**
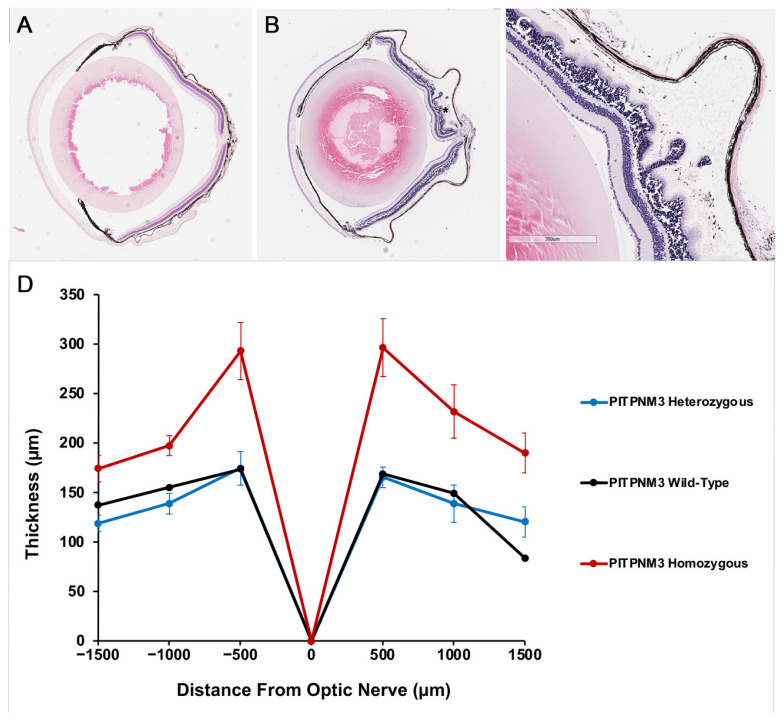
Retinal histology shows retinal detachment and photoreceptor degeneration in PITPNM3 mutation 1 year after introduction of mutation. (**A**) Histology from a PITPNM3 heterozygous, RD1 wild-type mouse line showing intact photoreceptor cells and organized retinal layers. (**B**) Histology from a PITPNM3 homozygous, RD1 wild-type mouse line with retinal detachment of the RPE (black asterisk), which was present in a subset of homozygous animals. (**C**) Histology from a PITPNM3 homozygous, RD1 wild-type mouse line with widespread photoreceptor degeneration and disorganization of the outer nuclear layer. Scale bar, 300 μm. (**D**) Spidergram shows mean retinal thickness (±SEM) for the three genotypes: wild-type (black line), heterozygous (blue line), and homozygous (red line) mice at six specified distances from the optic nerve head. Please note that increased retinal thickness in homozygous mice is driven by pathological changes, such as retinal detachment and subretinal fluid, which artifactually elevate thickness measurements despite the presence of marked photoreceptor loss. Heterozygotes show preserved lamination and thickness comparable to wild-type controls.

Mice ERGs were evaluated in scotopic (Figure 2A), photopic (Figure 2B) and maximum (Figure 3B) conditions. In an ANCOVA model adjusting for age, mutant PITPNM3 mice exhibited significantly larger photopic a wave amplitudes compared to controls (mutation *p* = 0.00024; Levene’s *p* = 0.0097), with both heterozygotes (*p* = 0.034) and homozygotes (*p* = 0.0002) showing diminished responses. Photopic b waves did not differ by genotype (*p* = 0.224) or age (*p* = 0.15). There was no significant difference by genotype by scotopic a wave (*p* = 0.892) and b wave amplitudes (*p* = 0.693), or by maximum a wave (*p* = 0.421) and b wave (*p* = 0.851). Age was not significant in all dark-adapted measures (all *p* > 0.14), and variance was equal across genotypes (Levene’s *p* > 0.11). The photopic a-wave differences indicate that PITPNM3 mutations selectively effect cone driven responses while rod driven and maximal ERG components are unchanged.

### 3.3. Histology

In the PITPNM3 mouse model, histological evaluation one year after mutation revealed discrete differences between genotypes. Sections from heterozygous animals displayed preserved photoreceptor cells and well-organized layers (Figure 3A), while Homozygous mutants occasionally exhibited retinal detachment of the RPE and widespread photoreceptor degeneration with outer nuclear layer disorganization. Retinal detachment was not observed in all homozygous animals, but in a subset of this group. (Figure 3B,C). Figure 3D shows retinal thickness measured at six locations, with no significant overall differences by PITPNM3 genotype. Post hoc Tukey-adjusted pairwise contrasts showed increased retinal thickness in homozygous mutants compared to heterozygotes (estimate = +87.7 μm, *p* = 0.0017); wild-type and heterozygous mice showed no statistical difference from each other. Notably, the increase in retinal thickness seen in homozygous mutants is not indicative of improved retinal health but instead reflects pathological enlargement due to chronic retinal detachment and accumulation of subretinal fluid. These pathological features artifactually increase measured thickness despite the actual loss of photoreceptors and structural disorganization, as shown in the representative histology panels. Heterozygotes consistently maintain normal lamination and thickness, matching the wild-type phenotype.

In the mouse model, the manifestation of disease was genotype-dependent. Heterozygous mutants maintained intact architecture, while homozygous PITPNM3 mutants developed retinal detachment and extensive photoreceptor cell loss after one year. Retinal detachment was seen only in a subset of homozygous mutants, not in all individuals of this group. ERG deficits in photoreceptor function preceded these histological changes, paralleling the clinical situation where functional loss may present before structural atrophy is visible. However, overall disease progression and severity were notably attenuated compared to human PITPNM3-associated retinopathy, highlighting limitations in recapitulating the full clinical spectrum within the preclinical setting.

Collectively, these findings demonstrate that the combined use of imaging, electrophysiological and histological techniques enables a detailed characterization of PITPNM3-related disease in both mice and humans. The in vivo mouse model shows early functional impairment followed by morphological degeneration predominantly in the homozygous genotype, though at a milder rate and reduced severity relative to human disease. These results underscore the complexity of modeling inherited retinal dystrophies and the necessity for extended follow-up and diverse experimental approaches to improve the translational value of preclinical studies.

## 4. Discussion

The differences observed in the mouse model compared with human phenotypes associated with PITPNM3 underscore the need for further investigation. The results of the study revealed subtle differences in the generated mouse model compared to human phenotypes associated with PITPNM3 mutations. Full-field electroretinogram (ERG) analysis indicated a reduced cone response in the heterozygous and homozygous mice, although the severity was not as pronounced as observed in humans with PITPNM3-related conditions. Histological examinations of selected cases showed no major morphological changes in the retinal structure. Ultimately, the preclinical mouse model generated showed weak genotype–phenotype concordance and does not fully recapitulate the spectrum of human phenotypes caused by PITPNM3 mutations. The study highlights the complex and nuanced nature of PITPNM3-related retinal disorders. There are several possible causes for the discrepancy between the mouse model and human PITPNM3 mutation associated conditions.

First, disease progression may differ between humans and mice, and this study had a relatively short follow-up period of one year. PITPNM3-related retinal degeneration is a progressive condition that develops over time; thus, a relatively short follow-up limits the ability to observe more pronounced phenotypic changes. Our study design included evaluation at 8, 16, and 52 weeks, which captured both early presymptomatic and later degenerative stages. Changes in ERG were seen at 8 and 16 weeks and substantial retinal degeneration was present by 52 weeks, as captured in Figure 3. The multi-time point approach therefore enabled us to map the progression of pathology and revealed that pronounced phenotypic changes in this model are delayed. In humans, PITPNM3 retinopathies can have decreased visual acuity years before changes are observed on fundus imaging and retinal thickness. In this study, the observed decreases in phototransduction seen on ERG indicate mild degeneration of rod and cone cells in the outer segment, the retina layer in mice where PITPNM3 is expressed, further supporting incomplete manifestation of PITPNM3 phenotype. Future studies should explore longer follow-up to additional functional and structural assessments to gain a more comprehensive understanding of the disease phenotype in the mouse model.

Second, using young animals in the preclinical model may have limited the biological variability or predictability of the observed phenotypic changes. Disease manifestations in humans with PITPNM3-related conditions are influenced by various genetic and environmental factors that may not be fully replicated in the mouse model. In RP, for example, non-genetic factors such as oxidative stress modulate disease progression. After rod cell loss in RP, oxidative stress increases; this has been suggested to play a role in rod and cone degeneration. Oxidative damage is markedly increased in the ocular samples of RP patients and anti-oxidant interventions substantially delay photoreceptor cell death, suggesting that oxidative stress is a key contributor to retinal degeneration in RP. Additionally, RP exhibits diverse heterogeneity in phenotype with young animals.

Lastly, mouse models, while valuable for studying genetic disorders, may not fully recapitulate the complexity of human diseases. Limitations include differences in retinal anatomy, cellular composition, and functional characteristics between mice and humans may contribute to the observed discrepancies in phenotype severity. Expression of PITPNM3 in mice retina is isolated to the outer nuclear segments, while expression of PITPNM3 in humans is highly expressed in the brain and spleen.

These findings highlight the importance of longer follow-up periods in preclinical studies to capture the full spectrum of disease progression and evaluate the long-term effects of PITPNM3 mutations. Additionally, the need to consider genetic variability and different genetic backgrounds in animal models is crucial for understanding the genotype–phenotype relationship. The discrepancies between the mouse model and human phenotypes suggest that caution should be exercised when translating findings from animal studies to human clinical applications. While animal models provide valuable insights into disease mechanisms and therapeutic interventions, the complexity and interplay of genetic and environmental factors in human patients cannot be fully replicated in animal models alone.

While our study contributes valuable insights into phenotype-genotype concordance in PITPNM3, there are several limitations that may impact the interpretation and generalizability of our findings. One limitation is the discrepancy in sample sizes of mice genotypes. This disparity can limit subgroup analyses as smaller sample size groups may not have sufficient statistical power. Additionally, RD1 retinal degeneration mutations in the mouse line are a confounding factor as contamination renders it difficult to isolate the study results to the effects of PITPNM3 mutation. This study also has a limited scope, exploring one PITPNM3 mutation variant, and may not be broadly applicable to other PITPNM3 mutations. Additional limitations include the relatively short follow-up period and use of young animals as previously described.

Future research should extend study durations, involve older animals, and consider diverse mouse strains to better replicate human conditions. The utilization of animal models necessitates meticulous justification, careful evaluation of alternatives, and an assessment of the associated benefits. Whenever feasible, exploring alternatives to animal models is advisable, such as utilizing Drosophila and investigating genetic variants in humans. Furthermore, investigating the impact of oxidative stress and employing alternative models like zebrafish or fruit flies could offer additional insights, contributing to a more holistic understanding of PITPNM3′s involvement in retinal degeneration. The incorporation of additional genetic variants, such as PDE6, which exhibits phenotypic similarities to PITPNM3, and the consideration of various disease stages, could provide complementary insights, leading to a more thorough comprehension of PITPNM3′s role in retinal degeneration.

## 5. Conclusions

This study’s development of a preclinical mouse model for PITPNM3-related phenotypes marks an important step in understanding the genotype–phenotype relationship in these disorders. Although the mouse model did not fully replicate the severity of human conditions, it provided valuable insights into the potential discrepancies in disease progression and phenotypic expression. Our findings stress the importance of further investigation, including studies with longer durations and the inclusion of environmental and age-related factors. A deeper understanding of PITPNM3-associated disorders is crucial for advancing personalized medicine and developing targeted treatments in the field of genetic ophthalmology.

## Data Availability

The raw data supporting the conclusions of this article will be made available by the authors on request.

## Abbreviations

The following abbreviations are used in this manuscript:

RP	Retinitis Pigmentosa
PCR	Polymerase Chain Reaction
RPE	Retinal Pigment Epithelium
FAF	Fundus Autofluorescence
OCT	Optical Coherence Tomography
SD-OCT	Spectral-domain optical coherence tomography
ERG	Electroretinogram

## Appendix A

### Appendix A.1. Electroretinogram (ERG) for Mouse Retina

The Electroretinogram (ERG) is an electrophysiological test adapted for mouse retinal studies, offering insights into the functionality of mouse photoreceptors and other retinal cells. ERG tests were administered to both eyes of all mice using previously described methods. ERG testing was performed 6, 8, and 16 weeks. Mice were dark-adapted for 12 h prior to anesthetization with 0.1 mL/10 g B.W. of 1 mL of 100 mg/mL ketamine and 0.1 mL of 20 mg/mL xylazine in 8.9 mL PBS, which was injected i.p. During anesthetization, mice were placed on heating pads to maintain their body temperature at 37 °C. Mouse eyes were dilated with one drop per eye of Tropicamide Ophthalmic Solution (1%; Akorn). Electrodes were placed on the corneas, and Gonak Hypromellose Ophthalmic Demulcent Solution (2.5%; Akorn) was applied to the eyes to prevent corneal scarring. ERG recordings were simultaneously for both eyes. Controlled light stimuli of varying intensities are then delivered in a darkened room, similar to the human procedure. The electrode captures the electrical responses of the mouse retina, while additional electrodes serve as references. The analysis of mouse ERG data still focuses on the amplitude and latency of the A-wave and B-wave within the ERG waveform, providing valuable information about the functional status of photoreceptors and inner retinal cells. This adapted technique is crucial for studying retinal conditions and assessing the impact of genetic or therapeutic interventions in mouse models.

ERG tests were administered to both eyes of all mice using previously described methods. (1,2,3) ERG testing was performed at 6, 8, and 10 weeks. Mice were dark-adapted for 12 h prior to anesthetization with 0.1 mL/10 g B.W. of 1 mL of 100 mg/mL ketamine and 0.1 mL of 20 mg/mL xylazine in 8.9 mL PBS, which was injected i.p., as previously described. 4 During anesthetization, mice were placed on heating pads to maintain their body temperature at 37 °C. Mouse eyes were dilated with one drop per eye of Tropicamide Ophthalmic Solution (1%; Akorn). Electrodes were placed on the corneas, and Gonak Hypromellose Ophthalmic Demulcent Solution (2.5%; Akorn) was applied to the eyes to prevent corneal scarring. ERG recordings were simultaneously for both eyes.

### Appendix A.2. Histological Analysis of the Mouse Retina

Histological analysis of the mouse retina offers a detailed examination at the cellular level, revealing structural and pathological changes. The process begins with the preparation of mouse retinal tissue through fixation, embedding, and sectioning, adapted to the smaller scale of mouse eyes. These retinal sections are then stained to differentiate various cell types and structures, enabling meticulous observation under a microscope. Histology is indispensable for understanding the pathophysiological mechanisms of retinal diseases in mice and evaluating the effects of experimental treatments. It allows researchers to visualize changes in retinal layers and cellular integrity in conditions like mouse models of AMD, retinal degeneration, and diabetic retinopathy, providing essential insights for both research and preclinical studies.
